# Dosing study of massage for chronic neck pain: protocol for the dose response evaluation and analysis of massage [DREAM] trial

**DOI:** 10.1186/1472-6882-12-158

**Published:** 2012-09-18

**Authors:** Karen J Sherman, Andrea J Cook, Janet R Kahn, Rene J Hawkes, Robert D Wellman, Daniel C Cherkin

**Affiliations:** 1 Group Health Research Institute, 1730 Minor Avenue, Suite 1600, Seattle, WA, USA; 2 Department of Epidemiology, University of Washington, Seattle, WA, USA; 3 Departments of Family Medicine and Health Services, University of Washington, Seattle, WA, USA; 4 Department of Biostatistics, University of Washington, Seattle, WA, USA; 5 Department of Psychiatry, University of Vermont College of Medicine, Burlington, VT, USA

**Keywords:** Therapeutic massage, Optimal dose, Chronic neck pain

## Abstract

**Background:**

Despite the growing popularity of massage, its effectiveness for treating neck pain remains unclear, largely because of the poor quality of research. A major deficiency of previous studies has been their use of low “doses” of massage that massage therapists consider inadequate. Unfortunately, the number of minutes per massage session, sessions per week, or weeks of treatment necessary for massage to have beneficial or optimal effects are not known. This study is designed to address these gaps in our knowledge by determining, for persons with chronic neck pain: 1) the optimal combination of number of treatments per week and length of individual treatment session, and 2) the optimal number of weeks of treatment.

**Methods/design:**

In this study, 228 persons with chronic non-specific neck pain will be recruited from primary health care clinics in a large health care system in the Seattle area. Participants will be randomized to a wait list control group or 4 weeks of treatment with one of 5 different dosing combinations (2 or 3 30-min treatments per week or 1, 2, or 3 60-min treatments per week). At the end of this 4-week primary treatment period, participants initially receiving each of the 5 dosing combinations will be randomized to a secondary treatment period of either no additional treatment or 6 weekly 60-min massages. The primary outcomes, neck-related dysfunction and pain, will be assessed by blinded telephone interviewers 5, 12, and 26 weeks post-randomization. To better characterize the trajectory of treatment effects, these interview data will be supplemented with outcomes data collected by internet questionnaire at 10, 16, 20 and 39 weeks. Comparisons of outcomes for the 6 groups during the primary treatment period will identify the optimal weekly dose, while comparisons of outcomes during the secondary treatment period will determine if 10 weeks of treatment is superior to 4 weeks.

**Discussion:**

A broad dosing schedule was included in this trial. If adherence to any of these doses is poor, those doses will be discontinued.

**Trial registration:**

This trial is registered in ClinicalTrials.gov, with the ID number of NCT01122836

## Background

Neck pain is a common health problem in the United States and other developed countries. An estimated 70% of adults are afflicted by neck pain at some time in their lives [1,2], 30 to 50% of adults are bothered by neck pain each year, [3] 10 to 15% of adults report neck pain that has persisted more than 6 months in the past year, [1,4] and 5% of adults are currently experiencing disabling neck pain [2]. A recent survey found that 25% of Canadians who had back and/or neck pain had sought care for their condition in the prior month, and that those with more severe pain were substantially more likely to seek care [5]. Another study found care seekers with chronic neck pain were most likely to visit primary care providers (72%) chiropractors (40%), physical therapists (35%), orthopedic surgeons (32%) and massage therapists (28%) [6]. Neck pain is the second leading health-related reason for time off work, after low back pain, [7] and millions of dollars are spent annually on treatment, lost wages, and compensation for neck pain [8,9].

As with back pain, a plethora of options are available for treating neck pain, yet the most commonly used treatments lack consistent evidence of effectiveness, especially for persons with chronic neck pain [6]. Standard medical practice employs rest, medication, physical medicine modalities, and education [10,11]. Medications, especially non-steroidal anti-inflammatories, and referral for physiotherapy were the most common treatments used by general practitioners in a recent study of how primary care physicians diagnose and treat patients with chronic neck pain [12]. Treatment effects, even for therapies demonstrating “benefit”, are typically small and it remains unclear if treatment benefits persist beyond 6 months [13].

With the paucity of proven conventional treatments, it is not surprising that neck pain is one of the top two conditions (second to back pain) for which American adults use CAM therapies, [14-16] most commonly chiropractic or massage. Almost 1 in 4 chiropractic visits and 1 in 5 massage visits are for neck symptoms [16,17] and only back pain is more commonly treated by these professions [17]. However, the effectiveness of massage for neck pain has not clearly been established. A recent systematic review evaluating the benefits of massage for mechanical neck disorders concluded that “the contribution of massage to manage cervical pain remains unclear” [18]. Only 6 of the 19 studies included in this review focused on massage as the sole treatment and only 4 of these 6 studies offered more than one treatment session. These 4 studies each examined a different type of massage and used a different treatment schedule. In fact, none of the 19 studies evaluated a type of massage that Americans would normally receive for neck pain. Given the generally low “dose” of massage, the heterogeneous treatment protocols, the frequent inclusion of other treatments, and the failure to study massage treatments for neck pain as they are currently provided in the U.S., it is not surprising that, despite 19 small trials, the benefits of massage remain unclear. The review recommended that researchers establish optimal dosing parameters for massage and test them in preliminary studies before undertaking large clinical trials [18]. A more recent review of 10 trials comparing massage to a control or other treatments for sub-acute and chronic neck pain concluded that massage was effective for relieving neck pain and related symptoms immediately post-treatment, but data on longer term outcomes were not available [19].

This study, designed to provide a firm foundation for more rigorous research on massage in the future and using a massage protocol that includes techniques that are used by massage therapists in the US, will determine the optimal minimal dose of therapeutic massage for chronic neck pain. We will evaluate three key components of optimal dosing: length of each treatment session, frequency of treatments per week, and the total treatment period. The specific aims are:

1. To determine the optimal combination of frequency of treatment and length of treatment

2. To determine the optimal duration of the treatment period

## Methods/design

### Overview

This trial was designed to determine the optimal dose of massage for persons with chronic non-specific neck pain. We conceptualize “dose” as including a specific weekly frequency of treatment, length of each treatment session and number of weeks of treatment. We conceptualize the “optimal dose” to be the minimal dose offering the most benefit but still having adequate adherence to treatment (i.e. at least 80% of participants in a dose attending at least 75% of the treatment sessions without serious adverse events possibly attributable to massage). In the trial, 228 study participants will be recruited and randomized to either a wait list control group or to 4 weeks of treatment with one of 5 different weekly dosing combinations (with 38 persons per group). At the end of 4 weeks (the primary treatment period), participants in each of the 5 different dosing combination groups will be randomized to receive either no additional treatment or to weekly 60-min massages for an additional 6 weeks (the secondary treatment period). Participants in the waitlist group will receive four weekly 60-min massages.

**Figure 1:**
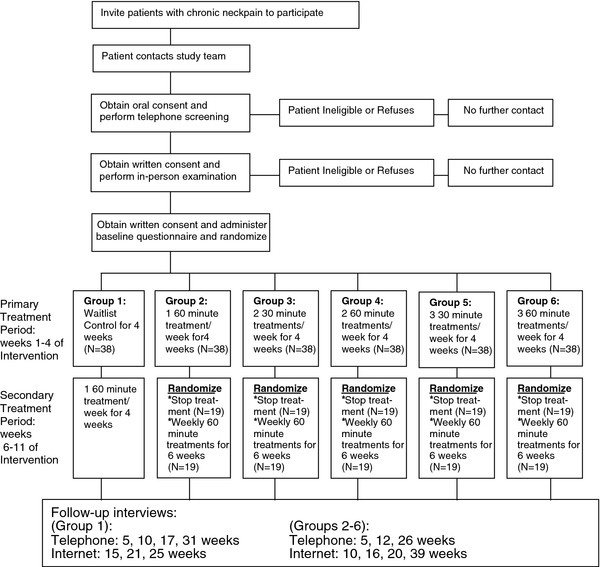
Participant focused flow chart.

Telephone interviewers unaware of treatment assignment will assess functional status and pain at 5 weeks post-randomization (end of primary treatment period) and again after 12 weeks (end of secondary treatment period) and 26 weeks. To more completely characterize the course of treatment effects, we will also collect data on the primary outcomes using internet questionnaires at 10, 16, 20 and 39 weeks. Figure 1 depicts the basic study design.

### Rationale for dosing schedule

We chose a primary treatment period of 4 weeks because our small randomized trial found [20] clinically important improvement among massage recipients by 4 weeks when receiving one 60 min massage per week. By having 5 groups receiving different doses over the first 4 weeks, we will be able to determine which weekly dose is optimal during this period. A secondary treatment period of 6 weeks was chosen because in our previous trial, up to 10 treatments were given over a period of 10 weeks. This will allow us to evaluate the benefits of an additional 6 weeks of treatment (with weekly 60-min doses).

This study design allows an examination of 5 doses that cover a broad range of feasible frequency/week (1, 2, 3 times) and treatment lengths (30 and 60 min). In order to test doses within the full range of likely beneficial doses, the dose range begins with the dose found effective in our pilot trial [20] (i.e., 60 min/week) and includes higher doses than those experienced massage therapists typically employ in their practices. We did not include 90 min visits because massage therapists were concerned that such a long treatment might aggravate the neck. By including doses that substantially exceed common practice, we can be more confident that we have probably included the “optimal dose”.

### Study population

Participants will be recruited from potentially eligible persons in the metropolitan Seattle area who receive care at Group Health, an integrated health care system that serves over 400,000 members through its own primary care facilities in Western Washington, and from the general population of the Seattle metropolitan area. Eligible individuals will have non-radicular uncomplicated chronic neck pain of non-specific origin. Neck pain due to infectious, inflammatory, spinal stenosis, and neoplastic causes, which are rare, will be explicitly excluded in this group of adults between 20 and 64 years of age.

### Inclusion and exclusion criteria

Inclusion and exclusion criteria were developed with the goal of maximizing enrollment of appropriate participants while screening out patients: who have neck pain of a specific (e.g. spinal stenosis) or complicated (e.g. due to a medical condition) nature, for whom massage is contraindicated (e.g. previous stroke), or whose medical conditions might make it difficult or impossible to receive the treatments (e.g. gross obesity or severe psychiatric conditions). These criteria (Table 1) are intended to exclude patients with medical conditions that: might contribute to an increased risk of a severe adverse event, would not allow for fully informed consent, or might lead to misinterpretation of the data (e.g. neuropathy of the hands because it might interfere with pain sensation).

**Table 1:** Inclusion and exclusion criteria

**Inclusion criteria**	**Exclusion criteria**
Between 20 and 64 years of age (Older people have higher risk of undiagnosed serious conditions causing neck pain)	Previous massage for neck pain in the last year or any massage in the last 3 months
Both men and women, any race or ethnicity	Neck pain lasting less than 3 months
At least one primary care visit for neck pain within the past 3 – 12 months	Mild symptoms and no functional impairment (less than 4 on a 0 to 10 pain intensity scale and less than 5 on the Neck Disability Index)
Non-specific, uncomplicated neck pain, i.e., ICD-9	Identifiable disease or condition could be the cause of neck pain (metastatic cancer or unexplained weight loss, discitis, disk herniation, vertebral fracture, infectious cause of neck pain, severe or progressive scoliosis, spondylolishthesis, spinal stenosis, osteoporosis)
codes: 723.1 Cervicalgia	
847.0 Cervical strain or sprain	
839.00-839.08 Cervical subluxation	Complicated neck problem (cervical radiculopathy, previous neck surgery, motor vehicle accident within past 3 months) Medico-legal issues (seeking or receiving compensation/litigation for neck or back pain)
722.4 Cervical intervertebral disc degeneration	
721.0 Cervical spondylosis without myelopathy	Other conditions that might confound treatment effects or interpretation of the results (e.g., disabling heart or lung disease, diabetic neuropathy, severe fibromyalgia, severe untreated depression, receiving treatment for hepatitis or AIDS, rheumatoid arthritis, planning to seek CAM or medical treatments for neck pain)
Symptoms consistent with non-specific, uncomplicated neck pain	
Lives or works within 45 minutes travel time from study clinic	Potential contraindications for massage (pregnancy, dizziness while lying supine, symptoms consistent with transient ischemic attack (TIA), previous stroke, hypersensitivity to touch or loss of sensation)
Give informed consent	Conditions making informed consent or treatment difficult (dementia, unable to read or speak English, paralysis, physically unable to undergo massage sessions – e.g., cannot get on/off massage table, major psychoses, severe problems with hearing or vision, lack of transportation, scheduling problems, plan to move out of town)

### Recruitment procedures and eligibility assessment

Recruitment of Group Health members will occur through the use of targeted mailings to members who have had visits to a primary care physician with a diagnosis indicating non-specific uncomplicated neck pain and through advertisements in Group Health’s quarterly magazine. We will also recruit participants from the general population of the Seattle metropolitan area through a variety of outlets including, but not limited to, the use of direct mail postcards, newspaper advertisements and posters in buildings in the downtown area.

Mailed letters explaining the study will contain the study’s “hot line” number and postcard on which persons interested in the study can write their names and contact information and mail back to our study staff in a postage-paid reply envelope. An interviewer will then phone prospective participants to answer questions and obtain verbal consent to determine provisional eligibility. Interviewers will use a computer program to guide them through a series of screening questions. The screening process will end with documentation in a database of either ineligibility or provisional eligibility.

Individuals found provisionally eligible will be invited to the clinic for an in-person exam. After obtaining consent, a trained nurse practitioner will examine the patient to confirm the presence of chronic non-specific neck pain and rule out cervical radiculopathy, dizziness while lying supine, or other conditions that may make massage inappropriate. The nurse will also confirm the absence of severe psychiatric conditions (i.e. a physician diagnosis of schizophrenia or bipolar disorder or severe depression), a motor vehicle accident within the last 3 months and symptoms consistent with transient ischemic attack (TIA). If confirmed eligible, a member of the study staff will make sure that the prospective participant understands the study requirements, administer informed consent following study procedures (including HIPAA), and answer any questions. To ensure consistency of mode of data collection, participants will then be taken to a nearby room where a research specialist will telephone the participant and administer the baseline questionnaire using a computer assisted interviewing program.

### Randomization to treatment group

A total of 228 participants with neck pain will be randomized (38 to each of the 6 study groups). After collecting baseline data, the Research Specialist will electronically randomize participants to treatment group. Treatment groups will be assigned by a computer-generated sequence of random numbers using a program that ensures that treatment allocation cannot be changed after randomization. Participants will be randomized in blocks of varying size to ensure balanced but unpredictable assignment of participants to all treatment groups. The computer will be programmed to randomize within two strata based on the baseline value of neck dysfunction as measured by the Neck Disability Index (5 to 14 and 15+).

After 4 weeks of treatment, individuals within each of the 5 treatment groups (Groups 2–6) who complete the 5 week outcome assessment will again be randomized, within group, to receive 6 more weekly 60 min sessions or no additional treatment. This will be done using independent block randomization, with stratification by baseline value of neck dysfunction, so that there are approximately equal numbers of participants in each treatment group randomized to additional treatment and to no additional treatment. This second randomization will allow us to evaluate the benefits, if any, of 6 weeks of additional treatment. Because Group 1 (wait list controls) had been promised massage treatment after the waiting period, they will receive weekly 60 min massages for 4 weeks.

### Massage protocol: dosing regimens

For the primary treatment period of four weeks, participants will be randomized to a wait list control or one of five different dosing regimens, which vary on the basis of the number of treatments per week (1, 2, or 3) and the length of the treatment visit (30 or 60 min). These regimens are:

1) 60 min of massage once a week

2) 30 min of massage twice a week

3) 60 min of massage twice a week

4) 30 min of massage three times a week

5) 60 min of massage three times a week

Thus, study participants will receive a total of 4 to 12 h of massage and a total of 4 to 12 treatments, depending on their group assignment.

### Massage protocols: common features

Distinct treatment protocols have been defined for both the 30 and 60 min massages to ensure that the massage therapist spends roughly the same proportion of time in each phase of treatment. Both protocols were designed with the expectation that the session would include roughly 60% of the treatment in the supine position and 40% in the prone position. However, the side-lying position may be substituted if appropriate. The assessment and massage techniques permitted (and prohibited) in all groups are identical. The protocols were based on the treatment protocol developed in our earlier study, [20] and were modified with the guidance of a panel of seasoned massage therapists and educators.

Assessment of all patients will include a detailed history of the neck complaint (part of which will be obtained prior to the treatment by the nurse practitioner) as well as active, passive, and resistive range of motion and palpation of the tissue. Range of motion will always be assessed just prior to treatment, and may be repeated during treatment at the therapist’s discretion. Tissue palpation can be incorporated into the treatment itself.

The following techniques are permitted as part of the treatment: application of cold; application of heat; compression; craniosacral techniques; direct pressure; friction; cross-fiber friction; effleurage (gliding); holding; petrissage (kneading); lymphatic drainage; muscle energy technique (MET) and proprioceptive neuromuscular facilitation (PNF); myofascial techniques; percussion (tapotement); positional release/strain-counterstrain; rocking, jostling, shaking, vibration; active, resistive and passive range of motion; stripping; traction; trigger point therapy.

There are required, recommended and prohibited components of the treatment itself. Required components are documented in the 30 and 60 min protocol descriptions. Recommended components include specific techniques such as holding, gentle petrissage or craniosacral work on the skull and face, addressing connective structures in the thoracic/chest and lumbar regions, and work on the upper extremities to address nerve tissue and vascular structures. Because this study is focused on the dose of soft-tissue manipulation, prohibited components include movement re-education, energetic techniques, background music, and self-care recommendations of any type. Other prohibited components of the protocol are designed to minimize adverse events. These include no shiatsu or other pressure point massage on the anterior neck, not overworking any region of the body (especially the cervical area), and avoiding any techniques that prompt splinting or bracing anywhere in the body. However, to facilitate a treatment that may be beneficial without causing increased pain, massage therapists are asked to discuss with each participant the level of pressure (i.e., a level that would cause ‘discomfort, but not pain) that would be appropriate during the treatment.

### Massage treatment protocol: 30 – minute visit

Massage therapists may modify any of the recommendations below based on safety of the participant. Other permitted techniques may also be used.

Recommended prototype session:

1) Cervical ROM Assessment (Active, Passive and Resistive ROM to be done in this order. Important to do this at outset to know the participant’s incoming limits. (approx. 3 min.)

2) Hands-on Check-in/tissue warming, etc. (1 min)

3) Lymph Drainage using recommended protocol (2 min)

4) Neck work [defined as skull thru upper back/chest, C7/T1, clavicles to 2nd/3^rd^ ribs & sternum – and everywhere the neck muscles insert]. (12–15 min). The following strokes are recommended; doing all of them is not required, nor is the precise order.

a. Friction on base of skull

b. Long strokes down lamina from base of skull – slow repetitive strokes with thumb on both sides of spine

c. Slow friction of the anterior neck muscles (not necessarily cross-fiber)

d. Slow friction to scalenes (other strokes to scalenes as appropriate)

e. Deeper longitudinal stripping techniques running parallel to muscle fibers to encourage muscle lengthening

f. Treatment of scar tissue wherever found (friction or myofascial techniques), along with areas affected by scar tissue. This is optional, based on therapist clinical judgment.

g. Effleurage and/or petrissage of the trapezius, paraspinals, splenius cervicus/capitus, levator scapula and sternocleidomastoid muscles as blending strokes between a-f above to ensure participants stay relaxed and for smooth transition between strokes.

h. Stretching to finish and enhance soft tissue manipulation – including PNF, MET, any active assisted stretching

5) Therapist to address compensatory patterns found in upper body, upper and lower extremities, pelvis, etc. (4–8 min) using supine, prone and/or side-lying positions as appropriate and effective for participant

6) Integration, which may also include some assessment to see how the session is holding (2–8 min). Possible strokes include:

a. Craniosacral techniques

b. stretching

c. rocking

d. other techniques as appropriate

7) Completion – (1 min)

### Massage treatment protocol: 60 – minute visit

Massage therapists may modify any of the recommendations below based on safety of the participant. Other permitted techniques may also be used.

Recommended prototype session:

1) Cervical ROM Assessment (Active, Passive and Resistive ROM to be done in this order. Important to do this at outset to know the participant’s incoming limits. (approx.3 min.)

2) Hands-on Check-in/tissue warming, etc. (1 min)

3) Lymph Drainage using recommended protocol (2–4 min)

4) Neck work [defined as skull thru upper back/chest, C7/T1, clavicles to 2nd/3^rd^ ribs & sternum – and everywhere the neck muscles insert]. (12–20 min). The following strokes are recommended; doing all of them is not required, nor is the precise order.

a. Friction on base of skull

b. Long strokes down lamina from base of skull – slow repetitive strokes with thumb on both sides of spine

c. Slow friction of the anterior neck muscles (not necessarily cross-fiber)

d. Slow friction to scalenes (other strokes to scalenes as well)

e. Deeper longitudinal stripping techniques running parallel to muscle fibers to encourage muscle lengthening

f. Treatment of scar tissue wherever found (friction or myofascial techniques) – treat ways that scar tissue is affecting tissue around it. This is optional, based on therapist clinical judgment.

g. Effleurage or petrissage of the trapezius, paraspinals (splenius cervicus/capitus), levator scapula and sternocleidomastoid muscles as blending strokes to ensure participants will stay relaxed and also to transition between other strokes.

h. Stretching to finish and enhance soft tissue manipulation – including PNF, MET, any active assisted stretching.

5) Therapist to address compensatory patterns found in upper body, upper and lower extremities, pelvis, etc. (15–20 min) using supine, prone and/or side-lying positions as appropriate and effective for participant

6) Neck Work, Part 2. Using easing and integrating strokes for the neck check in to see that benefit achieved during the first neck work is reinforced and maintained. This work can be done before or after the full body integration. (6–10 min).

7) Integration, which may also include some assessment to see how the session is holding (8–18 min). Possible strokes include:

a. Craniosacral techniques

b. stretching

c. rocking

d. other techniques as appropriate

8) Completion – (1–2 min).

### Study massage therapists

All study treatments will be performed by licensed massage therapists in the research clinic at the Group Health Research Institute in rooms with massage tables. The therapists will need to have at least 5 years experience treating musculoskeletal pain, including neck pain and be comfortable strictly following the treatment protocol. Therapists will be recruited from within the network of alternative providers contracted to provide services to Group Health members, from the massage therapists who have previously worked on our studies, and by referral from trusted colleagues. In addition all massage therapists will have completed special hands-on training on the protocol and on the performance of massage in a research setting, including applicable regulations. They will also practice the protocol prior to the study to ensure that they are able to deliver each protocol in a comfortable and accurate manner.

### Treatment schedules and adherence

To monitor adherence to the treatment protocol during the study, a Research Specialist who is also a massage therapist will observe a randomly selected 5% of the visits and complete a checklist for each of those visits. Checklists will be reviewed for adherence to the protocol and any necessary corrections with the massage therapists will be made as the study proceeds.

### Natural history cohort study

In addition to inclusion of a 4-week wait list control group, we will recruit a separate cohort of 50 patients with chronic neck pain, who will serve as a natural history comparison group for the 9 month follow-up period. We decided to include a separate natural history group because we did not believe we could recruit and retain individuals randomized to a 9 month control group in the context of the dosing trial, where all other groups would received from 4 to 18 h of massage within the first 12 weeks of the study. We will recruit members for the natural history cohort who meet the same inclusion and exclusion criteria as the dosing study participants over the same time period. However, the natural history cohort will be recruited from the Puget Sound region by selecting zipcodes outside of the dosing study recruitment region centered in Seattle. We will recruit participants using the same procedures we use for the dosing trial but we will modify our telephone eligibility screening questions to reflect the lesser requirements of participating in the Natural History Control Group (i.e., we will not ask about availability for massage treatments, transportation to our downtown clinic location, ability to get on and off of a massage table, use of a wheelchair, etc.). We will also not require an in-person pre-randomization visit. After obtaining oral consent and collecting baseline data we will follow cohort members for 9 months asking them to complete the series of telephone interviews and internet questionnaires given to members of Groups 2 to 6. These questionnaires will be virtually identical to those of the trial participants, but will omit all references to adverse events and other effects of the study treatments.

### Assessment of outcomes

We will assess a set of key outcomes for neck pain adapted from recommendations made by the Philadelphia Panel [7] and the Cochrane review of physical treatments of neck pain [21]. These include neck specific disability, neck pain, patient global assessment, quality of life, days of neck-related restricted activity and work status, and patient satisfaction with care. We will also collect data on sociodemographic characteristics, neck pain history, treatment-related information and on co-interventions as summarized in Table 2 and described below.

**Table 2:** Content of baseline and follow-up questionnaires

**Measure**	**Baseline (telephone)**	**5-week (telephone)**	**12-week (telephone)**	10, 16, 20, and 39 wks** (internet)	**26-week (telephone)**
**BASELINE CHARACTERISTICS**					
Sociodemographic characteristics	x				
Neck pain history & current episode	x				
Expectations of treatment	x				
Knowledge of and experience with massage	x				
**CORE SET OF RECOMMENDED OUTCOMES FOR NECK PAIN STUDIES**					
*Neck Disability Index (dysfunction)	x	x	x	x	x
* Pain intensity	x	x	x	x	x
General Health Status (SF-36)	x	x^╪^			
Neck – related disability days in prior week	x	x	x		x
Satisfaction with care for neck problem	x	x	x		
Work Status	x	x	x		x
Patient Global Rating of Improvement		x	x		x
**TREATMENT-RELATED INFORMATION**					
Adverse experiences attributed to treatment		x	x		
Home practice recommendations		x	x		
**POTENTIAL CO-INTERVENTIONS**					
Use of medication for neck pain	x	x	x		x
Neck exercises	x	x	x		x
Use of other co-interventions for neck pain		x	x		x
**OTHER MEASURES**					
Perceived Stress	x	x	x		x

* Co-primary outcome measures. Groups 2–6 and natural history controls only; Group 1 will have additional phone interviews at 10, 17, 31 weeks.

** Internet questionnaires will be completed by participants in Groups 2–6 and the natural history study on this timeframe. Group 1 will complete internet questionnaires at 15, 21, and 25 weeks. ^╪^Group 1 only.

#### Baseline characteristics

Baseline information will be collected on the sociodemographic characteristics of study participants, neck pain history, and on the current episode of neck pain. We will also obtain participants' ratings of their expectations of the helpfulness of therapeutic massage for their neck pain. The SF- 36 measure, [22] recommended for neck pain studies by Gross et al. [21] will be used to measure general health status at baseline. This 36-item instrument measures functional status in eight domains and yields physical and mental health status summary scores. Because we have consistently found the SF-36 unresponsive to change in our populations of musculoskeletal pain, we will only administer it at the baseline interview.

#### Primary outcomes

Primary outcomes will be measured at 5, 12 and 26 weeks by telephone and at 10, 16, 20 and 39 weeks by internet survey. We will use the Neck Disability Index (NDI), [23] a 10-item questionnaire that was adapted from the popular Oswestry Index for back pain, to assess neck pain and dysfunction. The NDI measured at 5 weeks will serve as our primary time outcome. The NDI’s psychometric properties are better characterized than those of other neck specific questionnaires, even though it has the possible disadvantage of combining assessments of pain and dysfunction, which are separate dimensions. Because the NDI requires reading six moderately long response options for each question, we will send participants copies of the NDI before we administer the baseline and follow-up interviews and also post a copy on a section of the Group Health Research Institute website that is accessible by study participants.

We will measure pain intensity using a 0 to 10 scale, where 0 represents “no pain” and 10 represents “pain as bad as it can be”. Participants will be asked at baseline and during all follow-up interviews to rate the average intensity of their neck pain during the previous week. Such scales are positively correlated with other measures of pain intensity and have demonstrated sensitivity to change [24].

#### Secondary and other outcomes

Several secondary outcomes will also be measured at 5, 12 and 26 weeks. Days of restricted activity will serve as a surrogate for disability and will be measured with 3 National Health Interview Survey questions about how many days in the prior week individuals spent over half the day in bed, home from work or school, or cutting down on usual activities due to illness or injury [25]. These questions will be asked concerning activity limitations specifically attributable to neck problems. We will capture work status by asking participants who are not students, homemakers or retired if they are employed at their usual job, on light duty or restricted assignment, paid leave/sick leave, unpaid leave, unemployed because of health reasons, unemployed for other reasons, or on disability [26]. We will ask participants to provide a global rating of improvement in their neck related dysfunction on a seven point scale ranging from “completely gone” to “much worse”. Finally, we will ask participants about their perceived stress, using the 10-item version of the Perceived Stress Scale (PSS), [27] the most widely used self-report measure of psychological stress. The PSS is a state measure that usually asks about stress experienced over the last month. Because the PSS is influenced by factors that vary, such as daily hassles, life events, appraisals and coping resources, [28] it should be sensitive to any improvements in life stress due to the massage intervention.

Treatment-related Information: At each clinic visit and at the 5 and 12 week follow-up interviews immediately after the primary and secondary treatment period, we will collect information on any adverse experiences. At the 12 week interview, we will ask participants if they had received home practice recommendations from their massage therapist, and if so, what they were. Such recommendations are prohibited by the treatment protocol.

Potential Co-interventions: At the 5, 12 and 26 week telephone interviews, we will ask participants about their use of specific types of medications in the last 7 days and whether use has changed since their previous interview. We will also ask participants about whether they have done neck exercises in the last 7 days, and if so on how many days and for how long on average. Visits to various types of care providers for neck pain will be captured on the follow-up telephone questionnaires.

### Schedule of assessments

We will measure short-term outcomes, using telephone interviewers masked to treatment group, at the end of the 4 week primary treatment period (5 week interview) and at the end of the secondary treatment period (12 weeks; Groups 2–6 and natural history controls only) to determine whether the 4- and 10-week course of treatments have had any benefit (Tables 2 and 3). We will also measure longer-term outcomes at 26 weeks (in Groups 2 – 6 and natural history controls) via telephone interviews to determine whether those benefits persist for at least 4 months post-treatment and if there is a difference in persistence by dose. We will ask participants in Groups 2 through 6 (and natural history controls) to complete an 11-question survey by internet at 10, 16, 20, and 39 weeks post-randomization. (A mailed option will be available to participants without internet access.) Because the 11-question surveys were short and relatively frequent, we thought an intenet format maximize our response rates at these time points. This will allow us to more precisely characterize the consistency and persistence of any treatment benefits of the initial dose as well as of the 6-week booster dose. Group 1, the waitlist control, will be treated from weeks 5 to 9 and after that, will have follow-up phone interviews at weeks 10, 17, and 31 corresponding to Groups 2–6 5, 12, and 26 week post-treatment follow-up times.

**Table 3:** Study calendar for individual patient activities

		**Week number**																									
**Schedule of treatments and assessments**	**Pre-study and randomization**	**Wk 1**	**Wk 2**	**Wk 3**	**Wk 4**	**Wk 5**	**Wk 6**	**Wk 7**	**Wk 8**	**Wk 9**	**Wk 10**	**Wk 11**	**Wk 12**	**Wk 13-14**	**Wk 15**	**Wk 16**	**Wk 17**	**Wk 18-19**	**Wk 20**	**Wk 21**	**Wk 22-24**	**Wk 25**	**Wk 26**	**Wk 27-30**	**Wk 31**	**Wk 32-38**	**Wk 39**
Wait list Control (Group 1)		y	y	y	y																						
Primary Treatment Period																											
(Groups 2-6)		z	z	z	z																						
Primary Treatment Period																											
(Group 1)							y	y	y	y																	
Secondary Treatment Period																											
(50% of Groups 2 - 6)							z	z	z	z	z	z															
Informed Consent																											
	x, y																										
Baseline Characteristics	x, y					y																					
Primary Outcomes	x, y					x, y					x, y	x			y	x	y		x	y		y	x		y		x
Secondary Outcomes	x, y					x, y					y	x					y						x		y		
Adverse Events						z					y	z															
Potential Co-interventions	x, y					x, y					y	x					y						x		y		
Other Measures	x, y					x, y					y	x					y						x		y		

Legend: y=Group 1; x=Groups 2-6 & Natural History Controls; z=Group 2 - 6 only;=Internet survey.

### Data collection, processing, and quality control

In Groups 2–6 and the natural history cohort, outcomes information will be collected from participants by telephone interviewers unaware of treatment group at baseline, week 5 (end of primary treatment period), week 12 (end of secondary treatment period), and week 26 (Table 3). Participants in Group 1 will be interviewed by telephone interviewers unaware of treatment group at baseline, week 5 (end of waitlist period), week 10 (end of primary treatment period), 17 (comparable to end of other groups secondary treatment period), and week 31. We will also ask participants in Groups 2 through 6 and the natural history cohort to complete a short internet survey at 10, 16, 20, and 39 weeks post-randomization. Participants in Group 1 will be asked to complete a short internet survey at weeks 15, 21, and 25 weeks. This will allow us to more precisely characterize the persistence of any treatment benefits of the initial dose as well as of the 6-week booster dose. Results of follow-up data from internet questionnaires or telephone interviews will not be seen by the massage therapists.

We will attempt to obtain outcomes data from all participants in the trial and the natural history cohort, including those who fail to appear for any treatments, those who subsequently discontinue treatments, those who discontinue enrollment in the health plan, and those who move away. Baseline and follow-up telephone interviews will be conducted using Computer-Assisted Telephone Interviews (CATI). At the 5, 12 and 26 week follow-up interviews, the interviewers will be unaware of the participants’ treatment assignments. To maintain assessor masking for questions relevant for all study participants, questions focused on the massage treatment (when relevant) will not be asked until all other questions have been completed and stored in the CATI database. Baseline interview data will be collected before randomization to treatment group.

We will collect information on recruitment, randomization, and treatment processes so we can report patient flow according to the CONSORT guidelines [29,30]. Specifically, we will record the number of invitation brochures sent, the number of responses received, the resolution of these responses (ineligible, refused, eligible and randomized, other), the number of participants in all groups who received at least one massage treatment, the number of visits made, the number of participants providing follow-up data by group at each follow-up, the number of participants completing the trial, and the number of withdrawals due to: ineffective treatment, adverse experiences, loss to follow-up, or other causes.

We will create weekly reports to monitor recruitment, visit adherence, and follow-up interviews for all time points. This will allow us to identify and resolve any difficulties that arise in the course of the trial.

For all visits, the massage therapists will use Visit Forms to record important clinical and research information. These forms will identify participants only by their initials and Study ID numbers. The Initial Visit Form will include clinically useful information such as primary and secondary problems identified, medical history, the assessments performed and their findings, treatment techniques used, and their anatomic location. The Initial and Follow-up Visit Forms will be similar, except only the former will include medical history while only the latter will include an assessment of progress since the last visit.

Specific quality control procedures for data collection will be developed during the start-up phase of the trial. These will include procedures to ensure that the trial is running smoothly (e.g., randomization is proceeding as planned, recruitment is on schedule, data collection forms are accurately entered into databases, the computerized interviewing system is storing data correctly). The computerized interviewing programs will contain range and logic checks. Prior to recruitment, all data systems will be tested with imaginary participants. Data will be examined for completeness using computer programs developed specifically for that purpose. In addition, we will test all analytic programs to ensure that the analyses are accurate.

Computer files with patient names will be password protected with access restricted to staff using this information to recruit patients, obtain follow-up data, and interact with any patients reporting adverse events. During treatment, clinic charts containing Visit Forms (identified by Study ID numbers and patient initials) will be stored in locked filing cabinets in the research clinic. After being audited to ensure adherence to the protocol, completed visit charts will have initials removed, and again stored in locked filing cabinets accessible only to study personnel.

### Protection of human subjects and assessment of safety

#### Protection of human subjects

The study was approved by Group Health’s institutional review board, the Human Subjects Research Committee (IRB Number 00000668), which serves as the ethics committee. The study will be carried out in compliance with the Helsinki Declaration

#### Adverse events

Participants will be asked about adverse experiences at each clinic visit and during the 5 and 12-week telephone interviews. We will define an adverse experience as any unfavorable and unintended sign, symptom or disease temporally associated with the use of the massage treatments that could reasonably be related to the procedure. Because massage has relatively short-term physiological effects, we will not report adverse events that begin more than two weeks after a participant’s final massage treatment (or more than 14 weeks after randomization for the usual care control group). In addition, we will capture information about any Serious Adverse Events (i.e., any adverse event occurring during treatment that results in any of the following outcomes: death, a life-threatening adverse event, inpatient hospitalization or prolongation of existing hospitalization, a persistent or significant disability/incapacity, a congenital anomaly/birth defect, or cancer) regardless of when they occurred.

#### Safety monitoring

This trial will be monitored for safety by an independent Data Safety Monitoring Body (DSM Body) comprised of a biostatistician, primary care physician and massage therapist. The DSM Body will evaluate the adverse-experience data we will provide them every 6 months to protect the safety of the study participants. However, any massage related Serious Adverse Events or deaths for any reason will be reported to the DSMB within 7 days of discovery. Based on the observed adverse effects of the treatment under study, the DSM Body will make recommendations on a regular basis to the PI and the Office of Clinical and Regulatory Affairs at the National Center for Complementary and Alternative Medicine (NCCAM) regarding continuation, termination, or other modifications of the trial.

#### Stopping rules

The trial will be stopped only if the Data Safety Monitoring Body (DSMB) believes there is an unacceptable risk of serious adverse events in one or more of the treatment arms. In this case, the DSMB could decide to terminate one of the arms of the trial or the entire trial. Based on our previous research and on the small number of case reports, we believe the risk of serious adverse events related to massage therapy is low.

We are not proposing to perform an interim analysis for efficacy. Since sample size in each group is small (n=38 per group), we will need the total sample sizes to determine optimal dosing. We will, however, monitor adherence to the treatment visit schedule for each of the study arms. The DSMB will review the data every 6 months, but there will only be one formal stopping rule for a given treatment due to adherence issues halfway through recruitment after approximately 12 months of recruitment. Should we find that treatment adherence is particularly poor for one or more arms (i.e., ≥ 50% of a group attended less than 75% of the required visits), we will drop that arm and create a new randomization protocol that randomizes individuals in equal proportions to the remaining arms of the trial. We plan to follow-up all participants who were enrolled before stopping a given treatment group so that their data can be used in the per protocol analyses if deemed appropriate.

### Study calendar

The study calendar (Table 3) displays the timing of the activities with each participant in the trial and the natural history cohort.

### Statistical issues

#### Sample size and the detectable difference

Our sample was chosen to ensure adequate power to detect a significant difference between at least two of the 5 groups receiving a massage treatment in the first 4 weeks of the trial (and not just adequate power to find a difference between one or more of the treatment groups and the waitlist control group). This reflects the primary goal of the trial: to compare the effects of different doses of massage on neck-related disability, and not just to determine if massage is better than no-additional treatment (as in the waitlist control). Our primary outcome is the binary measure of clinically relevant improvement of at least 5 points on the Neck Disability Index (NDI) at 5 weeks compared to baseline NDI score [31]. Based on findings in our pilot study, we assumed that 7% of the waitlist control group and 35% of Group 2 (60-min weekly treatment) would improve. We lack pilot data to estimate improvement rates in the other groups. Therefore, to permit sample size estimation, we will assume that treatment benefits will increase with the number of treatments given per week (for a fixed weekly dose) and with the number of minutes of massage per week. We assume a two–fold improvement from the lowest to the highest weekly dose (i.e., 60 to 180 min), based on the expectations of our consultant massage therapists. Table 4 summarizes our assumptions for both the NDI and neck bothersomeness score.

**Table 4:** Assumptions for sample size calculations

**Group**	**Amount of massage (weekly dose)**	**Proportion with clinically relevant improvement on NDI (at least 5 point improvement)**	**Proportion with clinically relevant improvement on Bothersomeness score (≥ 30% improvement)**
1	Waitlist	0.07	0.10
2	60 min 1×/week (60 min)	0.35	0.48
3	30 min 2×/week (60 min)	0.40	0.55
4	60 min 2×/week (120 min)	0.55	0.65
5	30 min 3×/week (90 min)	0.60	0.70
6	60 min 3×/week (180 min)	0.70	0.80

To protect against multiple comparisons, we will use Fisher’s protected least significant difference approach. Although this approach has been shown to inadequately protect against the type I error rate for studies with more than 3 groups, [32] we ran several simulations to test the type I error rate for our specific study design of 6 groups, with a binary outcome. We did this for sample sizes ranging from 30 to 40 and assuming an overall baseline improvement rate of either 0.07 or 0.50. We found that the type I error remained below 0.05 for all these situations. Following Fisher’s protected least significant approach, we will first use the omnibus Chi-Square test to test if there is any significant difference between all 6 groups and, if we find one, we will test for pairwise differences between groups. With 34 participants per group, we will have 97% power to find a significant difference between at least two of the 6 groups and 80% power to find a significant difference between Group 6 and Group 2, the treatment groups assumed to differ most in proportion of improvement. Assuming 10% loss to follow-up (as found in our pilot study and in our studies of back pain), we plan to recruit 38 participants in each group, for a total sample size of 228 in the trial.

Our other primary outcome is improvement in the Neck Pain Intensity Score of at least 30% from baseline, which reflects clinically important improvement [33]. With a final sample size of 34 participants in each group we would have 99% power to find a significant difference between at least two of the 6 groups and 80% power to find a significant difference between Group 6 and Group 2 assuming improvement rates as outlined in Table 4. Therefore, we will have adequate power to assess both our primary outcomes.

It should be noted that both primary outcomes (function and symptoms) will be tested at each time point at the 0.05 level because they address separate scientific questions. Analyses of both outcomes at all follow-up times will be reported, imposing a more stringent requirement than simply reporting a sole significant [34].

To address our second aim, the optimal duration of the treatment period, we will assess whether continuing treatment for another 6 weeks (60-min weekly massages for 6 weeks) improves outcomes in Groups 2–6. To simplify the power calculations, we focus on the 12 week response outcome and present those here. For the NDI (as a binary outcome), the power is 81%, when adjusting for group, assuming there are 34 participants in each of the 5 groups and assuming that, compared with those whose treatment stopped at 4 weeks, the additional proportion of each group improving at least 5 points from baseline after receiving 10 weeks of treatment is 0.2. The power is 88% for the binary neck pain intensity outcome, when adjusting for group and assuming that there are 34 patients in each of the 5 groups and assuming that, compared with those whose treatment stopped at 4 weeks, the additional proportion of each group improving at least 30% from baseline after receiving 10 weeks of treatment is 0.2.

Our power calculations are based on simple comparisons of the follow-up scores at a single point in time with adjustment for baseline values using logistic regression. We also plan to adjust for other baseline characteristics (e.g., age, gender). Inclusion of such baseline covariates can improve precision of the variance estimate and therefore increase power. For analyses of follow-up data for weeks 10 through 39 we will use generalized estimating equation methodology to handle within person correlation, possibly increasing our power at a given time point.

In summary, we have excellent power to detect clinically meaningful differences for both primary outcomes at both 5 and 12 weeks.

#### Statistical analysis

The primary purpose of this trial is to assess the “optimal minimal dose” -- the minimal dose offering the most benefit while having adequate adherence (i.e., at least 80% of participants attending at least 75% of the treatment sessions) and no serious adverse events possibly attributable to massage. We will analyze the benefit of each “dose” for 3 overlapping subsets of our total number of study participants. Our primary analysis will be intention to treat and thus will include data for all participants, regardless of their treatment adherence. We will also conduct a pre-specified subgroup analysis for participants attending at least 75% of their treatment sessions. We will then conduct two per protocol analyses using the total hours of massage participants actually received over the four weeks, regardless of specific group. The first per protocol analysis will include all randomized treatment groups. This analysis may include the observations from the waitlist control group after they receive treatment to increase power as long as their outcomes after receiving treatment are shown to be similar to group 2 who were randomized to receive 60 min of massage once per week. The second per protocol analysis will also include the natural history control arm. We will first compare the natural history control arm and the waitlist control group at 5 weeks to make sure that this non-randomized control arm has comparable outcomes compared to the randomized waitlist group. We will make the comparisons adjusting for baseline characteristics. If the waitlist control group and the natural history control group are comparable we will conduct per protocol analyses including the natural history control group. Both per protocol analyses are exploratory analyses.

##### Primary treatment period: primary study outcomes at 5 weeks

**Figure 2:**
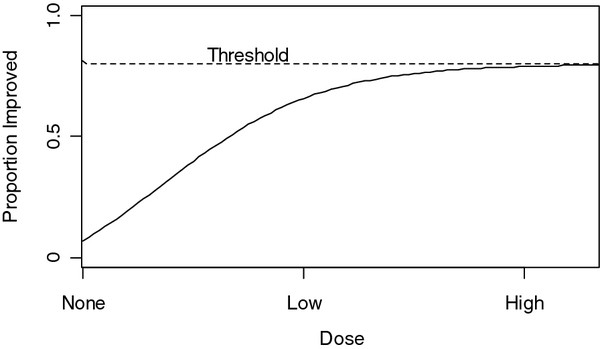
Standard dose response curves with a threshold effect.

The primary analyses will look at binary outcomes: 1) improvement of at least 5 points from baseline Neck Disability Index score and 2) improvement of at least 30% from baseline on the pain intensity score. We will use the following logistic regression model,

(1.1) $$\;\begin{array}{cc} \text{logit}\left(Y_{i}\right) & =\beta_{0}+\beta_{1}Group2_{i}+\beta_{2}Group3_{i}\\ & \;+\beta_{3}Group4_{i}+\beta_{4}Group5_{i}\\ & \;+\beta_{5}Group6_{i}+\alpha {Ζ}_{i} \end{array}$$

where *GroupA*_*i*_ is an indicator variable of 1 if participant i was randomized to group A or 0 otherwise (A=2, 3, 4, 5, or 6) and Z_*i*_ is a vector of potential confounders including baseline outcome score. We will use the Fisher’s protected least significant approach to assess if there are any differences among the 6 Groups and then, if there is a significant difference, to assess if any pairwise differences are significant. It is also of interest to run a parametric model to evaluate a trend over the weekly dose while taking into account the number of visits. In standard dosing analyses, at the lower doses, the proportion of patients improves with increasing dose, but as the dose increases to some higher level, further improvement is attenuated and approaches a threshold (see Figure 2).

In our study, it is not readily obvious what would be defined as a high dose or a low dose. Is a high dose determined simply by total dose or is it influenced by the number of visits given in a week? Our sample size calculations assume that dosing is a combination of both total dose and the number of visits. Because our design is a partial factorial design we are not able to run the full model including an interaction between cumulative dose and an indicator variable for each number of doses given. We can, however, evaluate the following model,

(1.2) $$\;\begin{array}{cc} \text{logit}\left(Y_{i}\right) & =\beta_{0}+\beta_{1}TotalDose_{i}+\beta_{2}NumVisits1_{i}\\ & \;+\beta_{3}NumVisits2_{i}+\beta_{4}NumVisits3_{i}+\alpha Z_{i} \end{array}$$

where *TotalDose*_*i*_ is the total number of minutes per week a participate is randomized to (e.g. Group 2 (60 min/week) is 60 min and Group 3 (30 min/2-week) is also 60 min), *NumVisitsB*_*i*_ is an indicator variable of 1 if participant *i* was randomized to a group with B visits per week or 0 otherwise (B=1, 2, or 3) and Z_*i*_ is a vector of potential confounders, including baseline outcome score.

**Figure 3:**
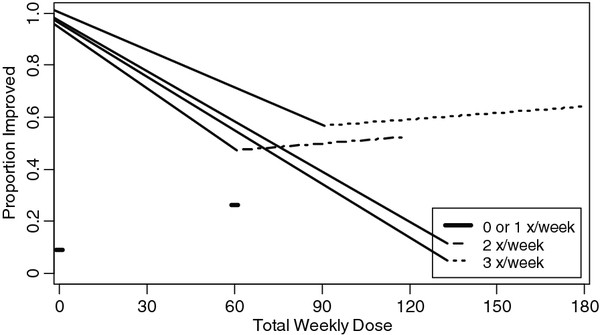
Plot of potential results from model 1.2 assessing the effect of total dose and number of weekly visits on proportion of improvement from baseline response.

Applying this model, we are able to assess, given a specific number of massages per week, if there is any significant linear trend in total dose. Because our dosing schedule includes multiple groups receiving either 2 or 3 visits per week (Groups 3 and 4 receive 2 doses per week and Groups 4 and 5 receive 3 doses per week), we will only be able to assess a linear trend of improvement in individuals in those pairs of groups. This would allow us to assess whether the number of doses, adjusted for total dose, is a significant predictor of outcome (*H*_*o*_: *β*_1_ = 0). Figure 3 displays an example of possible results from Model 1.2.

Model 1.1, the main effects model including all indicator variables, allows us to determine which groups differ from each other in terms of overall improvement. However, we have relatively low power to show a significant difference between groups 4, 5, and 6. Therefore, a failure to find a significant difference between groups 4, 5, and 6 could reflect a lack of power or a true absence of additional benefit (i.e., a thresholding effect; see Figure 2). Therefore we will also run the following model, not adjusting for number of visits, to evaluate a general trend of total dose,

(1.3) $$\;\log it\left(Y_{i}\right)=\beta_{0}+\beta_{1}TotalDose_{i}+\beta_{2}TotalDose_{i}^{2}+\alpha Z_{i}$$

If the quadratic term is not statistically significant then this would indicate no thresholding effect. We will also run more flexible models, such as splines, to assess an overall thresholding effect for benefit [35].

We will apply the same models specified above for our secondary analyses, but will use linear regression models for the continuous outcomes.

##### Secondary treatment period 2: primary study outcomes after 12 weeks

In evaluating the benefits of continuing treatment during the secondary treatment period, we will use generalized estimating equation (GEE) methodology to account for the correlation among a study participant’s repeated measures. We will look for changes from baseline in our primary outcomes to the longer term time points of interest (i.e., Week 12 and 26). We will also conduct secondary analyses looking at continuous outcomes using change scores from baseline to the time point of interest (i.e., Week 12 and 26). Because Group 1 (the waitlist control) will receive treatment during this period, the analyses will be restricted to Groups 2 through 6. The first model will include all main effects of primary dose (i.e., Group), additional treatment (i.e., Booster), and week as indicator variables:

(2.1) $$\begin{array}{l} \text{logit}\left(Y_{\mathrm{ij}}\right)=\beta_{0}+\beta_{1}Group3_{i}+\cdot \cdot \cdot +\beta_{4}Group6_{i}\\ \;+\beta_{5}Booster_{i}+\gamma_{1}Week26_{\mathrm{ij}}+\alpha Z_{i}\\ \text{for i=1,}..\text{,n and j=1,}2 \end{array}$$

where *Booster*_*i*_ is 1 if study participant *i* is randomized to continue treatment (i.e., 60 min 1×/week for 6 weeks) at week 5 and 0 otherwise, and *Week*26_*ij*_ is 1 if the outcome measurement was taken at Week 26 for study participant *i* at repeated measure *j* and 0 otherwise. In model 2.1 we will also incorporate potential interactions between Group, Booster, and Week, but we will have the most power to test for the main effects for Group and Booster and interactions with Week.

This model framework will allow us to answer certain key questions. For instance, we will be able to determine whether additional treatment beginning at week 5 improves outcomes at later time points (*H*_o_: *β*_5_ = 0) and if this improvement changes from week 12 to week 26 (i.e., a significant interaction between Week and Booster). We would also be able to assess if there are differences between the 5 Groups at week 12 and at week 26 and how these differences change over time. We will have less power to explore which Group by Booster dose is optimal, but we will at least be able to observe trends. This analysis will allow us to evaluate the relationships among Group, Booster treatment, and time. Further analyses will incorporate primary outcome data collected at follow-up time points via mail questionnaires (10, 16, 20, 39 weeks).

In summary, this study design including both the primary and secondary treatment periods, will allow us to assess the effect of different doses of massage, of different treatment durations (4 versus 10 weeks), and how these effects change over time and interact with each other.

## Discussion

Several logistical complexities are associated with this trial. Because there was little information about dosing available in the literature when this trial was designed, we used a rather broad dosing schedule, ranging from 60 to 180 min of treatment in 1, 2 or 3 weekly sessions. We anticipated that recruiting participants who are able to attend any of these dosing schedules could be challenging as could adherence in the highest doses. If necessary, such arms would be dropped. Another challenge was that persons with more mild neck pain might exaggerate their pain so that they could enter the trial. By not publicizing all entry criteria and by requiring a clinic visit before randomization, we plan to minimize these concerns.

## Competing interests

The authors have no competing interests to declare.

## Authors’ contributions

KJS, AJC, JRK, RAD, DCC participated in the conception of the trial and in plans for data analysis. RDW participated in plans for data analysis. KJS, AJC, RJH, RDW and DCC drafted the manuscript. All authors read and approved the final manuscript.

## Pre-publication history

The pre-publication history for this paper can be accessed here:

http://www.biomedcentral.com/1472-6882/12/158/prepub
